# Public Mental Health: Entwicklung eines Forschungs- und Handlungsfeldes

**DOI:** 10.1007/s00103-023-03681-9

**Published:** 2023-03-30

**Authors:** Joseph Kuhn, Peter Brieger, Martin Härter, Steffi Riedel-Heller

**Affiliations:** 1 GP 1 Gesundheitsberichterstattung, Epidemiologie, Sozialmedizin, Bayerisches Landesamt für Gesundheit und Lebensmittelsicherheit, Veterinärstr. 2, 85764 Oberschleißheim, Deutschland; 2 kbo-Isar-Amper-Klinikum, Vockestr. 72, 85540 Haar bei München, Deutschland; 3 Institut und Poliklinik für Medizinische Psychologie und Institut für Psychotherapie (IfP), Universitätsklinikum Hamburg-Eppendorf, Martinistraße 52 (W26) I, 20246 Hamburg, Deutschland; 4 Institut für Sozialmedizin, Arbeitsmedizin und Public Health, Universität Leipzig, Philipp-Rosenthal-Straße 55, 04103 Leipzig, Deutschland

Ein Drittel der Bevölkerung leidet im Laufe eines Jahres an einer klinisch relevanten psychischen Störung. Bei den Arbeitsunfähigkeitstagen geht inzwischen fast ein Fünftel auf psychische Störungen zurück. Sie stehen an erster Stelle bei den Frühberentungen und allein die direkten Krankheitskosten psychischer Störungen betragen fast 50 Mrd. € jährlich. Die psychische Gesundheit ist zweifellos ein Public-Health-Thema.

Dennoch hat die psychische Gesundheit der Bevölkerung unter Public-Health-Gesichtspunkten lange Zeit ein Schattendasein geführt. Der Blick war primär auf das psychisch kranke Individuum gerichtet. Das öffentliche Interesse bestand bestenfalls darin, gegenüber „störenden“ Individuen die gesellschaftliche Normalität durchzusetzen, im Notfall auch mit der zwangsweisen Unterbringung in einer psychiatrischen Klinik. Die Klinik vor den Toren der Stadt hat nicht nur die Kranken aus dem Sichtfeld der Gesellschaft geschafft, sondern auch die gesundheitspolitische Beschäftigung damit. Auch die Forschung war darauf konzentriert und ist es vielfach noch immer, individualmedizinische Fragestellungen zur Genese und Behandlung psychischer Störungen zu untersuchen.

Public-Health-Fragen, z. B. zu gesellschaftlichen Determinanten psychischer Gesundheit oder zur Gestaltung des Präventions- und Versorgungssystems, wurden in Deutschland lange Zeit kaum gestellt. In gewisser Weise könnte man hier die Antipsychiatriebewegung verortet sehen, als Bürgerrechtsbewegung gegen die Bevormundung psychisch kranker Menschen. Ansätze einer stärker systemischen Betrachtung gingen z. B. mit den verschiedenen vom Bundesministerium für Bildung und Forschung (BMBF) langjährig geförderten „Kompetenznetzen“ (z. B. zu Depression oder Schizophrenie) oder neuen Konzepten der integrierten Versorgung psychischer Störungen einher. Auch die vielerorts erstellten „Psychiatriepläne“ gaben zuweilen Impulse in dieser Richtung. Aber darüber hinausweisende grundlegende Bestandsaufnahmen und Reformperspektiven gab es kaum, abgesehen vom „Bericht über die Lage der Psychiatrie in der Bundesrepublik Deutschland“ im Jahr 1975, der berühmten „Psychiatrie-Enquête“ mit ihrem Fokus auf Enthospitalisierung und Aufbau gemeindepsychiatrischer Strukturen.

Eine Rolle mag dabei gespielt haben, dass psychische Störungen seit jeher Ausgrenzungsimpulse ausgelöst haben, gegenüber den betroffenen Menschen ebenso wie gegenüber dem Thema an sich. In Deutschland kommt zudem eine besondere historische Bürde hinzu. Sozialmedizin und Sozialhygiene sind im Nationalsozialismus von helfenden zu vernichtenden Konzepten umfunktioniert worden. Der Massenmord an psychisch Kranken haftet der Psychiatrie bis heute als historischer Makel an. Darüber hinaus hat die Pervertierung öffentlicher Gesundheit durch den Nationalsozialismus in der Bundesrepublik lange nachgewirkt, bis dahin, dass der Begriff „Volksgesundheit“ wohl dauerhaft vergiftet ist. Eine bevölkerungsorientierte Sozialmedizin konnte erst spät, in den 1980er-Jahren, wieder Fuß in Deutschland fassen, zunächst unter dem Begriff der „Public Health“. Schließlich beginnen die Gesundheitsämter erst jetzt wieder eine stärkere Rolle in der regionalen Gesundheitspolitik zu spielen.

In den letzten 20 Jahren lässt sich jedoch ein schrittweiser Wandel in der öffentlichen Wahrnehmung und Entstigmatisierung psychischer Störungen sowie eine Hinwendung zur allgemeineren Thematik psychischer Gesundheit feststellen. Während beispielsweise noch in den 1990er-Jahren den Versuchen, im Arbeitsschutzrecht die Prävention psychischer Belastungen auch expressis verbis zu verankern, massiver Widerstand seitens der Arbeitgeber entgegengebracht wurde, ist psychische Gesundheit heute ein zentrales Thema der „Gemeinsamen Deutschen Arbeitsschutzstrategie“. In der schon in den 1980er-Jahren aufkommenden Diskussion um Gesundheitsförderung als Leistung der Krankenkassen stand die psychische Gesundheit stets auf der Agenda, weil der stetig steigende Anteil psychischer Störungen am Krankenstand nach Lösungen verlangt hat. Auch ansonsten ist das Thema in der präventionspolitischen Landschaft angekommen. So hat beispielsweise die Nationale Präventionskonferenz im November 2022 einen Handlungsrahmen „Psychische Gesundheit im familiären Rahmen“ veröffentlicht – eine wichtige Brücke zu den durch das Präventionsgesetz vermittelten Initiativen.

In der Versorgung hat das Psychotherapeutengesetz 1999 neue Perspektiven eröffnet; die Weltgesundheitsorganisation hat in mehreren Strategiepapieren „Mental Health“ zur gesundheitspolitischen Herausforderung des 21. Jahrhunderts erklärt. Die Gesundheitsberichterstattung des Bundes und der Länder hat begonnen, die bevölkerungsweite Krankheitslast psychischer Störungen samt den relevanten Risikofaktoren zu vermessen, beginnend mit dem Bundesgesundheitssurvey 1998. Das „Aktionsbündnis Seelische Gesundheit“ bündelt eine breite Akteurslandschaft. Die Gesellschaft insgesamt ist bei der Entstigmatisierung psychischer Störungen trotz aller noch bestehenden Defizite große Schritte vorangekommen.

Wenn man so will, ist das Thema psychische Gesundheit auch hierzulande aus seinem historischen Schatten getreten. Es wird für bevölkerungsmedizinische und gesellschaftstheoretische Reflexionen zugänglicher. Unterstützend dabei waren auch Impulse von der europäischen Ebene. Beispielsweise hat der von der Europäischen Kommission 2008 auf den Weg gebrachte „Europäische Pakt für psychische Gesundheit und Wohlbefinden“ mit seinen prioritären Handlungsfeldern „Psychische Gesundheit von Kindern und Jugendlichen“, „Prävention von Depression und Suizid“, „Psychische Gesundheit im Alter“, „Psychische Gesundheit am Arbeitsplatz“ sowie „Stigma und soziale Ausgrenzung“ dezidiert gesellschaftliche Herausforderungen des Themas jenseits der unmittelbaren klinischen Versorgung in den Vordergrund gerückt. Spätere Initiativen sind dieser Linie gefolgt. Im Januar 2023 hat die Europäische Kommission ein Konsultationsverfahren für eine Initiative unter dem Arbeitstitel „Eine umfassende Herangehensweise an die psychische Gesundheit“ in die Wege geleitet, die bisherige Programme zusammenführen und auch neuere Herausforderungen wie die Coronakrise und den Ukrainekrieg aufnehmen soll.

Seit einiger Zeit wird am Robert Koch-Institut eine Nationale Mental Health Surveillance (MHS) aufgebaut. Sie wird ebenfalls dazu beitragen, das Handlungsfeld unter Public-Health-Gesichtspunkten auszuleuchten. Dafür wiederum sind Impulse aus der Praxis ebenso wie aus den Wissenschaften, die sich mit psychischer Gesundheit beschäftigen, notwendige Grundlagen. Vor dem Messen kommt das Denken: Was macht die Spezifität psychischer Störungen aus, wie lassen sie sich epidemiologisch erfassen, wie ist die Versorgungsstruktur aufgestellt, welche Präventionsansätze gibt es, welche Dimensionen sind darüber hinaus im Handlungsfeld „Public Mental Health“ zu beachten, etwa was gesellschaftliche Teilhabe angeht, Stigmatisierungen oder ethische Fragen?

Zu einigen dieser Fragen soll das vorliegende Heft Bestandsaufnahmen und Perspektiven liefern, auch als Fortsetzung des Schwerpunktheftes 2/2019 über Kooperationen bei der Versorgung psychischer Störungen. Das Themenfeld Public Mental Health ist sehr breit und kann hier nur exemplarisch präsentiert werden. Wichtige andere Aspekte, z. B. die psychische Gesundheit von Menschen mit Migrationshintergrund, die psychische Gesundheit in der Arbeitswelt oder Digitalisierungsfragen rund um dieses Thema, sind zum Teil schon in früheren Heften aufgegriffen worden oder werden es noch in späteren Heften. Wir hoffen, den Leserinnen und Lesern mit der vorliegenden Auswahl dennoch eine Orientierung in einem sich schnell wandelnden Themenbereich geben zu können und danken den Autorinnen und Autoren für ihre anregenden Überlegungen.

Dr. Joseph Kuhn

Prof. Dr. Peter Brieger

Prof. Dr. Dr. Martin Härter

Prof. Dr. Steffi Riedel-Heller MPH

